# Retrospective multicenter evaluation of oral step-down versus intravenous carbapenem treatment of extended-spectrum beta-lactamase-producing *Escherichia coli* urinary tract infections

**DOI:** 10.1017/ash.2026.10794

**Published:** 2026-07-24

**Authors:** Margarita Bellon, Alice Margulis Landayan, Jorge Morales, Jorge Murillo, Stephen Breazeale, Timothy P. Gauthier

**Affiliations:** 1 Department of Pharmacy, South Miami Hospital, South Miami, USA; 2 Department of Medicine, South Miami Hospital, South Miami, USA; 3 Baptist Health Academics, Baptist Health: Baptist Health South Florida Inc, Miami, USA; 4 Clinical Pharmacy Enterprise, Baptist Health: Baptist Health South Florida Inc, Miami, USA

## Abstract

**Objectives:**

This study evaluated whether treatment with oral step-down therapy compared with intravenous carbapenems alone for extended-spectrum beta-lactamase (ESBL)-producing *Escherichia coli* urinary tract infections (UTIs) impacted patient venous catheter days and clinical outcomes.

**Methods:**

This multicenter retrospective study at six different hospitals in a large health system included adults (≥ 18 years) admitted for ≥ 24 hours with ≥ 1 documented UTI symptom, a ceftriaxone-resistant *Escherichia coli* urine culture, and receipt of ≥ 1 dose of meropenem or ertapenem between August 2022 and July 2025. The primary outcome was median venous catheter line days directly associated with antibiotic use inpatient and outpatient. Secondary outcomes included pharmacist antibiotic intervention, incidence of antibiotic-related adverse events, hospital length of stay, duration of therapy, inpatient catheter line days, projected outpatient catheter line days, presence of peripherally inserted central catheter or midline, central line-associated bloodstream infection, and treatment failure (defined as escalation of antibiotics, in-hospital mortality, urgent care/emergency department visit or readmission, or recurrence of symptomatic UTI with ESBL *Escherichia coli* within 30 days).

**Results:**

120 patients were included, with 60 in the oral step-down and carbapenem groups, respectively. Primary outcome of median venous catheter days in the oral step-down group was 0 (IQR 0–0) vs 5 (IQR 0–7) in the carbapenem group, *p* < .001. Secondary outcome of treatment failure in the oral step-down group was 2 (3%) vs 8 (13%) in the carbapenem group, *p* < .09.

**Conclusions:**

Oral step-down therapy for ESBL *Escherichia coli* UTIs was associated with a significant reduction in venous catheter line use.

## Introduction

Urinary tract infections are one of the most common causes of hospitalization in adults in the United States, with *Escherichia coli* (*E. coli*) being the most common causative pathogen.^1^ Extended-spectrum beta-lactamase (ESBL) (eg, ceftriaxone-resistant) *E. coli* is currently recognized by the Centers for Disease Control and Prevention (CDC) as a serious threat to public health due to resistance to multiple antibiotics and increasing prevalence.^2^ Carbapenems (eg, meropenem, ertapenem) are generally regarded as the drugs of choice for treatment of ESBL infections.^3^ However, carbapenems are broad spectrum antibiotics with increased potential for collateral damage compared to oral (PO) step-down therapy.^3^

Despite the 2025 Infectious Diseases Society of America (IDSA) Guidelines for the Management of Complicated Urinary Tract Infections recommending transitioning to PO step-down therapy when susceptibilities allow, there is still some provider hesitance with this type of de-escalation due to concerns about treatment failure and recurrence.^4^ Per the 2024 IDSA Guidelines for the Management of Antimicrobial-resistant Gram-negative Infections, PO agents such as nitrofurantoin, fluoroquinolones, fosfomycin, and trimethoprim-sulfamethoxazole may be considered for ESBL *E. coli* cystitis if susceptibility is confirmed.^3^ In the setting of ESBL *E. coli* pyelonephritis, oral options are limited due to concerns for penetration to the kidneys with remaining potential oral options of fluoroquinolones and trimethoprim-sulfamethoxazole.^3^ Patients who are transitioned to oral step-down therapy experience shorter lengths of stay, similar clinical outcomes, and decreased costs.^5,6^ Another important consideration is that when patients are discharged on intravenous (IV) antibiotics, either a midline or peripherally inserted central catheter (PICC) line is placed for outpatient IV access, increasing risk of adverse effects such as catheter-associated bloodstream infections (CLABSI).^7^ Strategies to reduce the placement of central venous catheters decrease CLABSI risk.^8^

The aim of this study was to evaluate the impact of oral step-down therapy on patient venous catheter days directly associated with antibiotic use and clinical outcomes compared to treatment with IV carbapenems alone for ESBL *E. coli* UTIs.

## Methods

This was a multicenter, retrospective, observational, comparative cohort study conducted across six hospitals in a large health system. Patients were identified through cross-referencing a report extracted from Cerner® and a microbiology susceptibility report from VigiLanz® (a pharmacy surveillance system) to identify patients who met inclusion criteria. Patients were assigned to two groups: those who received IV carbapenem therapy only for their course of treatment versus those who were transitioned to PO step-down therapy for ESBL *E. coli* UTI treatment. Patients were eligible for study inclusion if they were adults (≥ 18 years) admitted for ≥ 24 hours with ≥ 1 documented UTI symptom (eg, dysuria, urinary urgency, frequent urination, flank pain), a ceftriaxone-resistant *E. coli* urine culture, and receipt of ≥ 1 dose of meropenem or ertapenem between August 2022 and July 2025. Exclusion criteria consisted of ineligibility for oral therapy (eg, no PO options based on susceptibilities and/or patient-specific factors, strict nothing per oral route (NPO), malabsorption syndrome, grade 3 or 4 mucositis, active gastrointestinal bleed, not taking any other PO medications), carbapenem-resistant *E. coli*, concomitant non-urinary infection sources (excluding bacteremia with same urinary pathogen), polymicrobial infection, prostatitis, chronic suppressive therapy, comfort or hospice care, pregnancy, and incarceration. Patients meeting inclusion criteria were evaluated over the course of their index admission and subsequent outpatient follow-up.

The primary outcome was median venous catheter line days directly associated with antibiotic use. The primary outcome was selected to capture the direct impact of oral step-down therapy on venous catheter exposure, an important stewardship target linked to catheter-associated complications, patient safety, and resource utilization. Secondary outcomes included pharmacist antibiotic intervention, incidence of antibiotic-related adverse events, hospital length of stay, duration of therapy, inpatient catheter line days, projected outpatient catheter line days, presence of PICC or midline, CLABSI, antibiotic acquisition costs, new resistance development, and treatment failure (defined as escalation of antibiotics, in-hospital mortality, urgent care/emergency department visit or readmission, or recurrence of symptomatic UTI with ESBL *E. coli* within 30 days), as well as the separate components of the composite outcome of treatment failure. New resistance development was defined as isolation, within 30 days of therapy completion, of the same ESBL-producing species from new cultures demonstrating resistance to one or more agents previously susceptible during the index admission. Antibiotic acquisition cost was collected as a descriptive economic measure to illustrate differences in drug purchasing costs between regimens, rather than as a prespecified clinical outcome.

During the study period, the health system utilized VigiLanz® to alert pharmacists to potential opportunities for de-escalation of antimicrobial therapy. The health system also has dedicated antimicrobial stewardship pharmacists who provide prospective audit and feedback. Pharmacist antibiotic intervention referred to actions performed by pharmacists, including but not limited to antimicrobial selection/change recommendations, dose optimization, and adjustment of treatment duration.

Continuous variables, including venous catheter line days, were assessed for normality. Continuous variables with non-normal distributions are reported as medians with interquartile ranges and were compared between groups using the Mann–Whitney U test. Categorical variables were summarized as counts and percentages and compared using the χ^2^ or Fisher’s exact test, as appropriate. All statistical tests were two-sided with a significance threshold of *p* < .05. A sample size of 51 participants per group (total *N* = 102) was calculated to achieve 80% power to detect a moderate effect size (Cohen’s *d* = .5) between two independent groups using a one-sided Student’s *t*-test at a significance level of .05. The study was approved by the Institutional Review Board of Baptist Health South Florida with a waiver granted for the requirement for informed consent and HIPAA authorization.

## Results

Of the 694 patients screened, 574 were excluded, with the most common reasons being no documented urinary symptoms (*n* = 297), ineligibility for oral options (*n* = 97), concomitant infections (*n* = 96), and presence of polymicrobial infections (*n* = 33). The final sample included 120 patients, with 60 in the carbapenem group and 60 in the PO step-down group. Baseline characteristics are described in Table 1. In the PO step-down group 31 (52%) patients were transitioned to PO therapy while inpatient. Median days of carbapenem therapy were 7 days (IQR 7–8) in the carbapenem group and 2 (IQR 2–3) in the PO step-down group, *p* < .001.

**Table 1.** Baseline Characteristics Table 1 long description.

Characteristics	Carbapenem group (*n* = 60)	Oral group (*n* = 60)	*P*-Value
Median age, years – (IQR)	80 (71–87)	77 (66–84)	.18
Male gender, *n* (%)	26 (43)	34 (57)	.06
Immunocompromised, *n* (%)	5 (8)	5 (8)	1.00
ICU admittance, *n* (%)	5 (8)	8 (13)	.38
Antibiotic allergies, *n* (%)^*^	18 (30)	16 (27)	.69
Penicillin	11 (18)	12 (20)	.82
Cephalosporin	4 (7)	2 (3)	.68
Sulfa	5 (8)	2 (3)	.44
Other antibiotic allergies	3 (5)	5 (8)	.30
Median creatinine clearance on admission, mL/min – (IQR)	47 (30–63)	46 (32–64)	.98
Symptomatic ESBL UTI in documented in the past 12 months, *n* (%)	12 (20)	13 (22)	.82
Uncomplicated UTI during current admission, *n* (%)	32 (53)	36 (60)	.46
Complicated UTI during current admission, *n* (%)	28 (47)	24 (41)	.46
Catheter-associated UTI	14 (23)	5 (8)	.43
* E. coli* bacteremia	1 (2)	1 (2)	1.00
Febrile UTI	4 (7)	7 (12)	.53
Other: UTI with systemic symptoms not categorizable as above	4 (7)	4 (7)	1.00
Pyelonephritis	5 (8)	7(12)	.54
UTI symptoms documented, *n* (%)^*^			
Dysuria	52 (87)	40 (67)	**.01**
Flank Pain	6 (10)	14 (23)	**.05**
Frequent urination	15 (25)	24 (40)	.07
Urinary urgency	6 (10)	11 (18)	.19
Outpatient antibiotic, *n* (%)^**^			
Nitrofurantoin	-	15 (25)	-
Trimethoprim-sulfamethoxazole	-	21 (35)	-
Fosfomycin	-	5 (8)	-
Levofloxacin	-	3 (5)	-
Meropenem	7 (12)	-	-
Ertapenem	33 (55)	-	-

* Patients may exist in more than one category.

** Not all patients were prescribed outpatient antibiotics.

ICU, intensive care unit; ESBL, extended-spectrum beta-lactamase; UTI, urinary tract infection; *E. coli, Escherichia coli*.

Primary outcome of median venous catheter days in the PO step-down group was 0 days (IQR 0–0) compared to 5 days (IQR 0–7) in the carbapenem group, *p* < .001. Secondary outcome of median antibiotic acquisition costs in the PO step-down group was $32.21 (IQR 19.67–55.40) versus $60.54 (IQR 44.94–77.13) in the carbapenem group, *p* < .001. Neither group experienced a CLABSI, new resistance following recurrence, or escalation of antibiotics. Additional secondary outcomes are described in Table 2.

**Table 2.** Secondary Outcomes Table 2 long description.

Secondary Outcomes	Carbapenem group (*n* = 60)	Oral group (*n* = 60)	*P*-Value
Pharmacist antibiotic intervention, *n* (%)	22 (37)	19 (32)	.56
Incidence of antibiotic-related adverse drug events, *n* (%)	1 (2)	1 (2)	1.00
Median inpatient line days, days – (IQR)	1 (0–2)	0 (0–0)	**<.001**
Midline placement, *n* (%)	27 (45)	1 (2)	**<.001**
PICC line placement, *n* (%)	13 (22)	0 (0)	**<.001**
Median projected outpatient line days – (IQR)	3 (0–5)	–	–
Median total duration of therapy for UTI treatment, days – (IQR)	7 (7–8)	8 (6–9)	.88
Median length of hospital stay, days – (IQR)	5 (3–8)	4 (3–5)	.08
Composite outcome of treatment failure, *n* (%)^*^	8 (13)	2 (3)	.09
Escalation of antibiotics	0 (0)	0 (0)	1.00
Hospital mortality within 30 days	0 (0)	0 (0)	1.00
Recurrence of symptomatic ESBL *E. coli* UTI within 30 days	4 (7)	0 (0)	.12
Urgent care, ED visit, or readmission within 30 days	8 (13)	2 (3)	.09

* Patients may exist in more than one category.

PICC, peripherally inserted central catheter; UTI, urinary tract infection; ESBL, extended-spectrum beta-lactamase; *E. coli, Escherichia coli*; ED, emergency department.

As the differences in key baseline characteristics such as age, sex, proportion of complicated infection, immunocompromised status, renal function, and intensive care unit (ICU) admission were not statistically significant between groups, further modeling to adjust for these covariates was not performed.

## Discussion

In this multicenter retrospective cohort of patients with ESBL *E. coli* UTIs, oral step-down therapy was associated with a statistically significant reduction in median venous catheter line days and antibiotic acquisition costs without worse clinical outcomes when compared to treatment with IV carbapenem therapy alone. By selecting venous catheter line days as the primary outcome, this study focused on a stewardship-relevant end point that directly reflects device exposure, risk of catheter-associated complications, and resource utilization, while evaluated secondary outcomes were selected to ensure that reducing catheter use did not compromise clinical effectiveness. Both groups were generally similar with respect to age, gender, immunocompromised status, baseline creatinine clearance, and distribution of uncomplicated versus complicated urinary tract infections, supporting the internal validity of the comparison. However, patients in the PO step-down therapy group reported a symptom profile that differed significantly from those in the carbapenem group, which may have affected provider perception of illness severity and, in turn, consideration for transition to oral therapy.

Although PO step-down therapy was the post-discharge plan for certain patients in the carbapenem group, their lengths of stay ultimately exceeded the duration of treatment, highlighting an area of opportunity for step-down during the inpatient stay. Notably, though not statistically significant, treatment failure occurred more frequently numerically in the carbapenem group; this finding should be interpreted cautiously. Carbapenems may have been preferentially selected for patients perceived to be at higher risk or with more complex illness, and such nuances are not fully captured by the retrospective severity markers available in our data set. These results are therefore hypothesis-generating and complement emerging literature suggesting that non-carbapenem β-lactams may be reasonable alternatives for certain ESBL-producing UTIs when appropriately selected and dosed.

The findings above are consistent with guideline-endorsed strategies that encourage PO step-down from carbapenems for ESBL-producing *E. coli* UTIs when susceptibilities and patient factors permit. Current IDSA guidance supports transition from carbapenems to oral agents such as fluoroquinolones or trimethoprim-sulfamethoxazole for ESBL *E. coli* UTIs when a susceptible option is available, with nitrofurantoin and fosfomycin reserved for cystitis.^3^ In our study, trimethoprim-sulfamethoxazole and nitrofurantoin were preferentially prescribed in comparison to fluoroquinolones, which reflects our local susceptibility patterns and provider comfort with these agents. Notably, rates of treatment failure remained low across these regimens, suggesting that appropriate selection among these oral options can preserve clinical outcomes while reducing venous catheter use and antibiotic acquisition costs.

Prior studies of PO step-down in ESBL *E. coli* UTIs have primarily focused only on specific agents or select populations and have similarly reported preserved efficacy with shorter hospitalization and lower costs.^5,6^ Veve et al.^5^ determined that fosfomycin was a reasonable outpatient or step-down alternative to ertapenem for ESBL *E. coli* UTIs, with comparable clinical outcomes and reduced healthcare utilization. Shi et al.^6^ demonstrated that trimethoprim-sulfamethoxazole step-down therapy allowed earlier discharge and decreased total costs among patients with ESBL UTIs. When compared to recent literature, this study adds multicenter data comparing PO step-down to IV carbapenem therapy specifically in ESBL *E. coli* UTIs and focuses on venous catheter days as a primary outcome directly linked to antibiotic usage and evaluating the potential for catheter-related harm.

These results highlight the need for continued education to support appropriate use of PO step-down therapy, reducing unnecessary IV exposure while maintaining therapeutic effectiveness. Additionally, longer follow-up periods may help monitor antimicrobial resistance patterns, particularly in patients with recurrent infections. Educational resources were developed and disseminated to drive change while evaluating resources that can be leveraged to prompt an increase in PO step-down therapy.

This study has several limitations. First, the retrospective design introduces the potential for selection bias, particularly regarding provider decisions to either continue IV carbapenem therapy versus transition to PO agents. Although we evaluated several markers of illness severity and complexity (including ICU admission, classification as complicated vs uncomplicated UTI, immunocompromised status, and renal function) and found no statistically significant differences between groups, unmeasured factors may still have influenced treatment selection and outcomes. Second, venous catheter line days were not normally distributed, which led us to summarize this primary outcome using medians with interquartile ranges and to employ non-parametric statistical tests. As a result, we did not report mean differences with 95% confidence intervals for catheter days, and linear regression modeling of this outcome was not pursued. These limitations highlight that our findings should be interpreted as observational and hypothesis-generating, rather than definitive causal estimates. Additionally, follow-up was limited to events captured within the health system; patients who sought care elsewhere may have experienced treatment failure or recurrence that were not identified. In regard to new resistance development, a 30-day window may underestimate longer-term resistance emergence, and results should be viewed as short-term observations only. Finally, as the study was conducted within a single large health system, local antimicrobial susceptibility patterns and prescribing practices may limit generalizability to other settings.

This multicenter retrospective observational study suggests that oral antibiotic step-down therapy for ESBL *E. coli* UTIs is associated with a significant reduction in venous catheter line use and costs without compromising clinical outcomes, supporting step-down strategies to optimize resources and promote stewardship. These findings in combination with existing guideline recommendations support the incorporation of PO step-down for ESBL *E. coli* UTIs into routine clinical practice.

## Table 1. Long description

The table compares baseline characteristics of patients in carbapenem and oral groups. It has 25 rows and 5 columns. The columns are labeled Characteristics, Carbapenem group (n = 60), Oral group (n = 60), and p-Value. The rows include various characteristics such as median age, gender, immunocompromised status, ICU admittance, antibiotic allergies, creatinine clearance, types of UTI, and symptoms documented. Each row provides specific values for both groups and a p-value indicating statistical significance. Notable trends include differences in dysuria and flank pain symptoms between the groups.

Navigate back to Table 1..

## Table 2. Long description

A table comparing secondary outcomes between the Carbapenem group and the Oral group, each with 60 participants. The table has 14 rows and 4 columns, including column headers and row labels. Column headers are Secondary Outcomes, Carbapenem group (n = 60), Oral group (n = 60), and p-Value. Row labels include various outcomes such as Pharmacist antibiotic intervention, Incidence of antibiotic-related adverse drug events, Median inpatient line days, Midline placement, PICC line placement, Median projected outpatient line days, Median total duration of therapy for UTI treatment, Median length of hospital stay, Composite outcome of treatment failure, Escalation of antibiotics, Hospital mortality within 30 days, Recurrence of symptomatic ESBL E. coli UTI within 30 days, and Urgent care, ED visit, or readmission within 30 days. Each row provides specific data points for both groups and the corresponding p-Value. Notable trends include significant differences in Median inpatient line days, Midline placement, and PICC line placement between the two groups.

Navigate back to Table 2..
